# Corneal Endothelial Safety and Intraocular Pressure Outcomes of MicroPulse Laser Trabeculoplasty in Treatment-Naïve Glaucoma: A Six-Month Retrospective Study

**DOI:** 10.3390/life16081237

**Published:** 2026-07-27

**Authors:** Ahmet Mehmet Somuncu, Ahmet Taner Uysal

**Affiliations:** Department of Ophthalmology, Trabzon Kanuni Training and Research Hospital, 61250 Trabzon, Türkiye; ahmettaneruysal@hotmail.com

**Keywords:** micropulse laser trabeculoplasty, glaucoma, intraocular pressure, corneal endothelium, specular microscopy, 577 nm laser

## Abstract

MicroPulse laser trabeculoplasty (MLT) lowers intraocular pressure (IOP) with subthreshold pulses that spare the surrounding tissue, yet its effect on the corneal endothelium is far less studied than that of selective laser trabeculoplasty, and almost unstudied in treatment-naïve eyes. We retrospectively reviewed 70 treatment-naïve glaucoma patients (one eye each) treated with a 577 nm yellow laser (Easyret^®^) in micropulse mode (300 µm, 300 ms, 1000 mW, 360°). Endothelial cell density, mean cell area, hexagonality, the coefficient of variation in cell size, and central corneal thickness were recorded by specular microscopy (SP-1P, Topcon), and IOP by Goldmann applanation, at baseline and 1, 3, and 6 months. Linear mixed-effects models used every available observation, so no patient was excluded for a missed visit. IOP fell from 25.9 ± 2.5 mmHg to 20.6 and 21.0 mmHg at one and three months (−5.32 and −5.00 mmHg, roughly 20%; both *p* < 0.001), then returned to 24.8 mmHg by six months, and the proportion reaching a 20% reduction fell from 62.5% to 4.6%. Endothelial cell density, mean cell area, and central corneal thickness held steady, and every morphological change stayed within the device’s repeatability limits on equivalence testing. MLT lowered IOP early but transiently; over six months no clinically meaningful change in corneal endothelial morphology was detected.

## 1. Introduction

Worldwide, glaucoma remains the leading cause of irreversible blindness, and an aging population keeps that burden climbing [1]. Only one thing is proven to slow the disease: lowering intraocular pressure (IOP), by drops, laser, or surgery [2]. Drops work, but patients struggle to keep to them and the side effects accumulate year on year. Laser is the alternative. In the LiGHT (Laser in Glaucoma and Ocular Hypertension) trial, selective laser trabeculoplasty (SLT) earned a first-line role: among newly diagnosed eyes it controlled pressure at least as well as drops, cost less over time, and remained safe across follow-up [3,4]. SLT targets the pigmented cells of the trabecular meshwork with brief, selective pulses, and avoids the coagulative scarring left by the older argon method [5,6,7].

MicroPulse laser trabeculoplasty (MLT) pushes that principle further. Instead of a single continuous burst, it chops the energy into short pulses separated by rest intervals, so the meshwork absorbs even less heat [8]. The 577 nm yellow wavelength fits the job well: the meshwork takes it up readily, while xanthophyll barely absorbs it. Across the first year its reported IOP reduction sits broadly alongside SLT, but with a lighter tissue footprint [9,10].

Because the cornea lies in the laser’s path, the endothelium is the layer that warrants attention. These cells do not regenerate; they spread to cover any loss rather than divide, so damage accumulates [11]. After SLT the endothelium shows only transient marks: a slight dip in cell count, a brief rise in cell-size variability, and dark spots on specular images that clear inside a month [12,13,14]. The MLT evidence is sparser, and most of it comes from medicated eyes, where prior drops and longer-standing disease blur the picture. Untreated eyes give a cleaner reading.

We therefore followed corneal endothelial morphology and IOP over six months in treatment-naïve glaucoma eyes given 577 nm MLT, with two straightforward questions in mind: does the procedure disturb the endothelium, and how long does the pressure reduction hold when no drops are added? Three features separate this work from most MLT reports. First, every eye was treatment-naïve and stayed off medication throughout, so drugs blur neither the pressure nor the endothelial reading. Second, we judged endothelial stability by formal equivalence against the device’s measurement error, not by the simple absence of a significant change. Third, we compared primary open-angle and pseudoexfoliation eyes head to head.

## 2. Materials and Methods

### 2.1. Study Design and Participants

We retrospectively reviewed treatment-naïve glaucoma patients treated with MLT at Trabzon Kanuni Training and Research Hospital between August 2022 and October 2025. The study adhered to the Declaration of Helsinki and had approval from the Trabzon University Faculty of Medicine Non-Interventional Clinical Research Ethics Committee (approval no. 2026/26186; meeting no. 2026/06; dated 15 June 2026).

**Figure 1:**
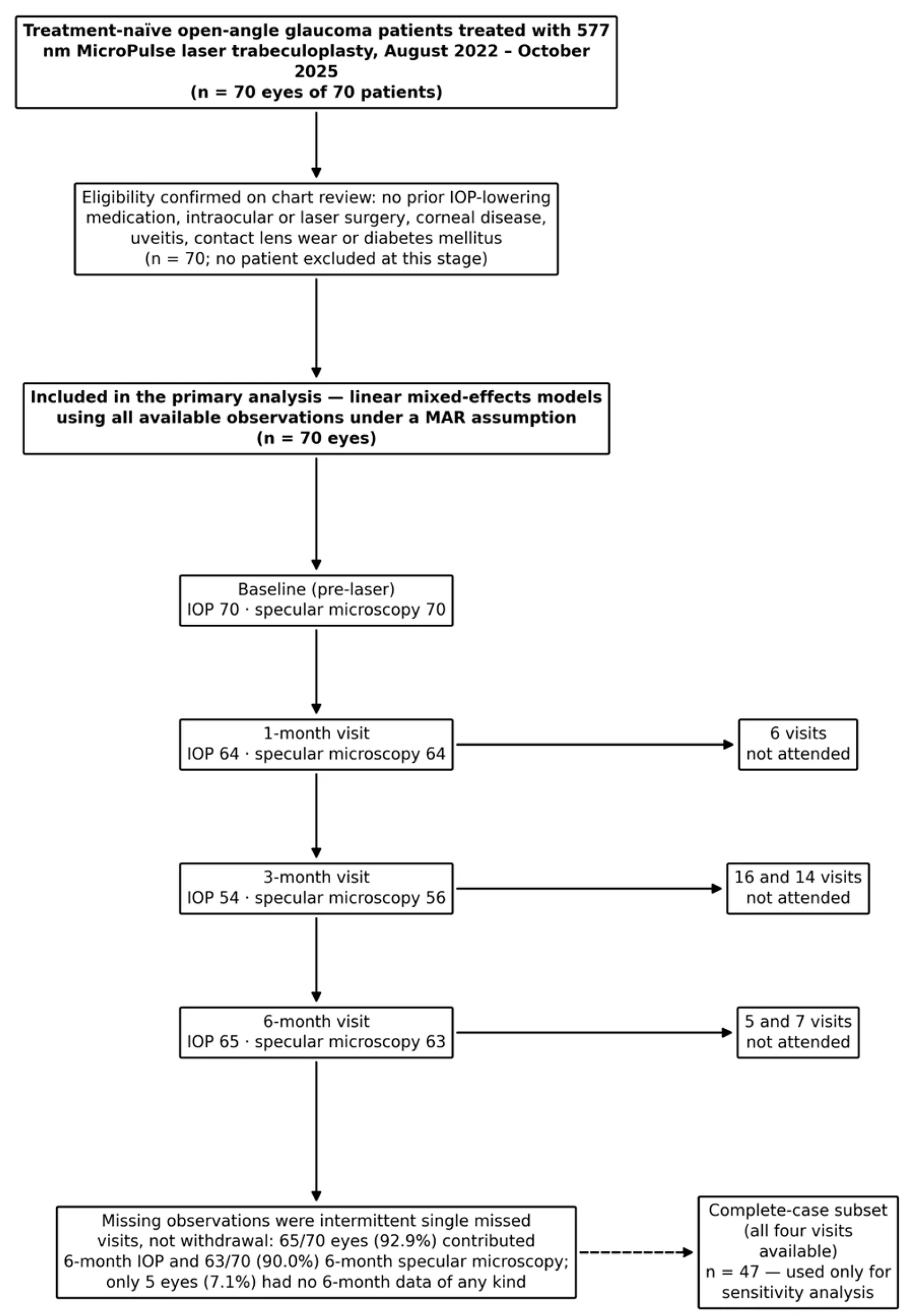
Flow of patients through the study. All 70 eyes entered the primary linear mixed-effects analysis; the numbers at each visit are the observations actually recorded, and the boxes on the right give the visits not attended. The complete-case subset (n = 47) was used only for sensitivity analysis. IOP, intraocular pressure; MAR, missing at random.

Eligible patients were adults with newly diagnosed, treatment-naïve open-angle glaucoma, whether primary open-angle (POAG) or pseudoexfoliation (PEX) glaucoma, who had never taken an IOP-lowering medication. Every eye had an open angle on gonioscopy (Shaffer grade 3 or 4). A history of intraocular or laser surgery, contact lens wear, corneal disease, uveitis, or diabetes mellitus was grounds for exclusion, since any of these can affect the corneal endothelium or its measurement on its own. Seventy patients met these criteria and underwent MLT during the study period (31 at a 10% duty cycle, 39 at 15%), and all seventy were carried into the analysis, one eye per patient. No patient was excluded for a missed follow-up visit; the handling of missing visits is set out in Section 2.4 and the flow of patients through the study in Figure 1. Throughout, IOP was taken with Goldmann applanation tonometry.

Both glaucoma subtypes were diagnosed on a uniform protocol applied to every patient in the cohort. All eyes underwent dilated slit-lamp biomicroscopy and gonioscopy, performed with Zeiss four-mirror and Goldmann lenses, by one of two ophthalmologists, both experienced glaucoma surgeons, before treatment. Pseudoexfoliation was recorded when characteristic greyish-white fibrillar material was seen after full pharmacological dilation at either of its two classic sites, the anterior lens capsule (a central disc, an intermediate clear zone, and a peripheral granular band) or the pupillary margin, with or without the associated features of iris sphincter transillumination defects, pigment dispersion onto the corneal endothelium and iris surface, and on gonioscopy heavy blotchy trabecular pigmentation or a Sampaolesi line anterior to Schwalbe’s line. Eyes were classified as POAG only when a fully dilated examination of both the lens capsule and the pupillary margin showed no exfoliative material and gonioscopy showed neither a Sampaolesi line nor the irregular heavy pigmentation typical of PEX. Subtype was assigned according to the findings in the treated (study) eye. In both subtypes the diagnosis of glaucoma itself required an untreated IOP above 21 mmHg together with a glaucomatous optic disc on slit-lamp biomicroscopic examination and optical coherence tomography. Because dilated slit-lamp examination and gonioscopy were performed and documented for every patient before treatment, subtype assignment was available for the whole cohort with no missing classifications.

### 2.2. Laser Protocol

A 577 nm yellow laser (Easyret^®^ yellow laser photocoagulator, Quantel Medical, Cournon-d’Auvergne, France) delivered MLT in micropulse mode, viewed through a Volk SLT gonioscopy lens (Volk Optical Inc., Mentor, OH, USA). Settings were a 300 µm spot, 300 ms exposure, and 1000 mW power across 360° of the trabecular meshwork, at a duty cycle of either 10% or 15%. The protocol pre-specified a comparison of these two settings, but in practice the choice followed operator preference: of the two surgeons, one routinely used 10% and the other 15%, so duty cycle and surgeon are fully confounded. Because no topical IOP-lowering drug was started at any point over the six months, the pressure values reflect MLT alone.

### 2.3. Specular Microscopy and Outcome Measures

A non-contact specular microscope (SP-1P, Topcon Corporation, Tokyo, Japan) imaged the corneal endothelium. All examinations across all four visits were performed by the same experienced ophthalmic technician on the same instrument, following a standardized acquisition protocol: the patient was seated and aligned on the chin rest, automated central-cornea captures were taken in auto-alignment mode without pharmacological dilation, the number of captures varying with patient cooperation, and the image with the largest countable cell area and no artifact was retained. Analysis used the instrument’s fully automated cell-analysis software. Specular microscopy was performed after applanation tonometry and before gonioscopy at each visit. Recorded were endothelial cell density (cells/mm^2^), mean cell area (µm^2^), the proportion of hexagonal cells (pleomorphism), the coefficient of variation in cell size (polymegethism), and central corneal thickness (µm) [15,16].

For this device the reported repeatability standard deviations are 70.7 cells/mm^2^ for endothelial cell density, 1.6 for the coefficient of variation, 4.9 percentage points for hexagonality, and 3.4 µm for central corneal thickness; the matching repeatability limits are 197.9 cells/mm^2^, 4.4, 13.7 percentage points, and 9.6 µm. These bounds let us judge whether a given change exceeded the instrument’s measurement error. They come from the device’s U.S. Food and Drug Administration (FDA) 510(k) clinical performance data in a separate cohort, and the repeatability of Topcon specular microscopy has been described before [17,18]; we therefore read them as external limits for measurement error rather than study-specific reliability estimates. Baseline and 1-, 3-, and 6-month visits used the same measurements plus IOP.

### 2.4. Statistical Analysis

We produced descriptive statistics in IBM SPSS Statistics version 26 (IBM Corp., Armonk, NY, USA), and fitted the mixed models and computed the equivalence tests, multiplicity corrections, effect sizes, and survival estimates in Python (3.12.3) (SciPy (1.17.1), statsmodels (0.14.6), and lifelines (0.30.3)). Continuous variables appear as mean ± standard deviation (SD). Each eye was measured at up to four visits, so the repeated structure was kept throughout.

Missing data were handled by analysis rather than by exclusion. All 70 patients entered the primary models, and every observation actually recorded was used. The primary tool for change over time was a linear mixed-effects model with a random intercept per patient and time as a categorical factor; under a missing-at-random assumption such a model uses all available data through maximum likelihood, so an eye that missed one visit still contributes the visits it attended. This preserves power and avoids the selection bias that listwise deletion would introduce. The model returned the mean change from baseline at each visit with 95% confidence intervals (CIs), and the overall time effect was tested by a Wald test on the three visit contrasts jointly. As a sensitivity analysis we repeated the whole analysis in the complete-case subset with all four visits available (n = 47), including the Friedman test as a distribution-free check; the two analyses are compared in the Results. To confirm that attendance was not related to baseline status, we compared complete-case and incomplete-case patients on every baseline variable.

Contrasts against baseline at 1, 3, and 6 months used the Wilcoxon signed-rank test on all available pairs, Holm-corrected within each parameter. IOP, treatment success, and the corneal-endothelial outcomes were handled as separate inferential families; within the corneal family, we further controlled the false discovery rate (Benjamini–Hochberg) across all fifteen comparisons. Effect sizes are Cohen’s dz. Rather than infer stability from a non-significant result, we tested it directly with two one-sided tests (TOST) for equivalence, setting each parameter’s repeatability limit as the equivalence margin. That limit is the largest difference attributable to measurement error alone, so any change inside it cannot be told apart from instrument noise; since this is a deliberately generous bound, we repeated every test against the stricter repeatability standard deviation as a sensitivity analysis. A change counted as equivalent to none when its 90% confidence interval sat entirely within ±margin. For each test we also looked at the achieved precision, meaning the tightest margin the data would still support. The Shapiro–Wilk test steered the choice of distribution-tolerant methods, and a two-sided *p* < 0.05 counted as significant.

Treatment success was set a priori as an IOP reduction of at least 20% from baseline, with no absolute pressure ceiling; we give the share of eyes meeting it at each visit among those examined at that visit, with Wilson confidence intervals. Durability is shown by a Kaplan–Meier curve in which failure was the first attended visit at which an eye no longer met the criterion. Eyes that still met the criterion when last seen, but were not examined at a later scheduled visit, were censored at that last visit with known status rather than excluded; because status was checked only at the three scheduled visits, the failure times are strictly interval-censored and the curve should be read as tracking visit times rather than continuous follow-up.

All comparisons between glaucoma subtypes were pre-specified as exploratory. Time-by-subtype interactions were tested within the mixed models and Holm-corrected across the six parameters, and the visit-wise between-subtype comparisons of change from baseline reported in Supplementary Table S1 were additionally subjected to Benjamini–Hochberg correction across all eighteen tests; both the unadjusted and adjusted values are reported so that the exploratory status of these analyses is explicit. Being a retrospective analysis of consecutive available cases, the study carried no a priori sample-size calculation.

## 3. Results

Seventy treatment-naïve patients contributed one eye each, and all seventy entered the analysis. Their mean age was 59.1 ± 5.3 years (range 47–72); 35 were women and 35 men, and right and left eyes were almost evenly split (36 right, 34 left). Thirty-nine eyes were treated at a 15% duty cycle and 31 at 10%. Primary open-angle glaucoma accounted for 48 eyes and pseudoexfoliation glaucoma for 22. Thirty-five patients had systemic hypertension and four had goitre, while the other 31 carried no systemic disease. None had prior ocular surgery, and none had corneal pathology. Baseline characteristics, for the cohort as a whole and stratified by glaucoma subtype, are given in Table 1.

Attendance was incomplete at the interim visits but the losses were scattered rather than systematic. IOP was recorded in 64 eyes at one month, 54 at three months and 65 at six months, and specular microscopy in 64, 56 and 63 eyes respectively; 47 eyes had all four visits. Crucially, the missing observations were single missed appointments rather than withdrawal: 65 of 70 eyes (92.9%) returned for the six-month IOP assessment and 63 of 70 (90.0%) for six-month specular microscopy, and only five eyes (7.1%) had no six-month data of any kind. Figure 1 sets out the flow. Patients with all four visits and those who missed at least one did not differ at baseline in age (59.3 ± 5.1 versus 58.7 ± 5.6 years, *p* = 0.59), IOP (26.0 ± 2.5 versus 25.5 ± 2.5 mmHg, *p* = 0.57), endothelial cell density (2605 ± 223 versus 2606 ± 231 cells/mm^2^, *p* = 0.95), mean cell area (*p* = 0.93), hexagonality (*p* = 0.44), coefficient of variation (*p* = 0.97), central corneal thickness (*p* = 0.88), sex, treated eye, glaucoma subtype or duty cycle (all *p* = 1.00; Supplementary Table S2). Attendance therefore looks unrelated to baseline status, which supports the missing-at-random assumption behind the mixed models.

**Figure 2:**
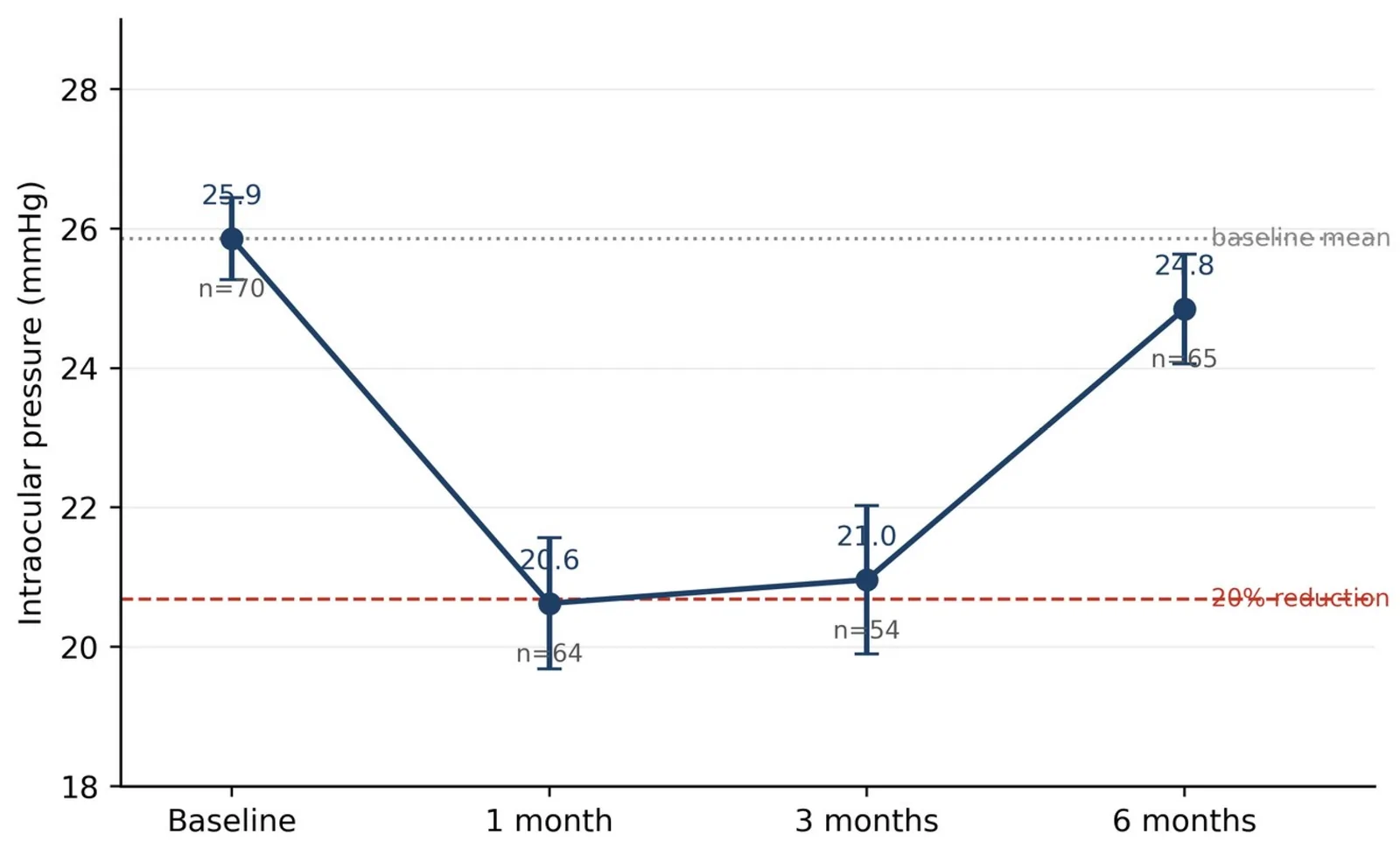
Mean intraocular pressure across visits after MicroPulse laser trabeculoplasty. Error bars, 95% confidence interval; dotted line, baseline mean; dashed line, the 20% reduction threshold. The number of eyes examined at each visit is shown beneath the corresponding point.

IOP responded fast. From a baseline of 25.9 ± 2.5 mmHg, IOP fell to 20.6 ± 3.9 mmHg at one month and 21.0 ± 4.0 mmHg at three. In the mixed model these came to reductions of −5.32 mmHg (95% CI −5.97, −4.67) and −5.00 mmHg (−5.69, −4.31), around 20% and highly significant (Holm-corrected Wilcoxon *p* < 0.001 at both visits; Cohen’s dz > 2). The gain proved short-lived. By six months mean IOP was back to 24.8 ± 3.2 mmHg, a mixed-model change of just −1.02 mmHg (−1.67, −0.37) or about 4% below baseline, and the joint Wald test confirmed a strong overall time effect (χ^2^ = 375.9, df = 3, *p* < 0.001; Figure 2, Table 2).

**Figure 3:**
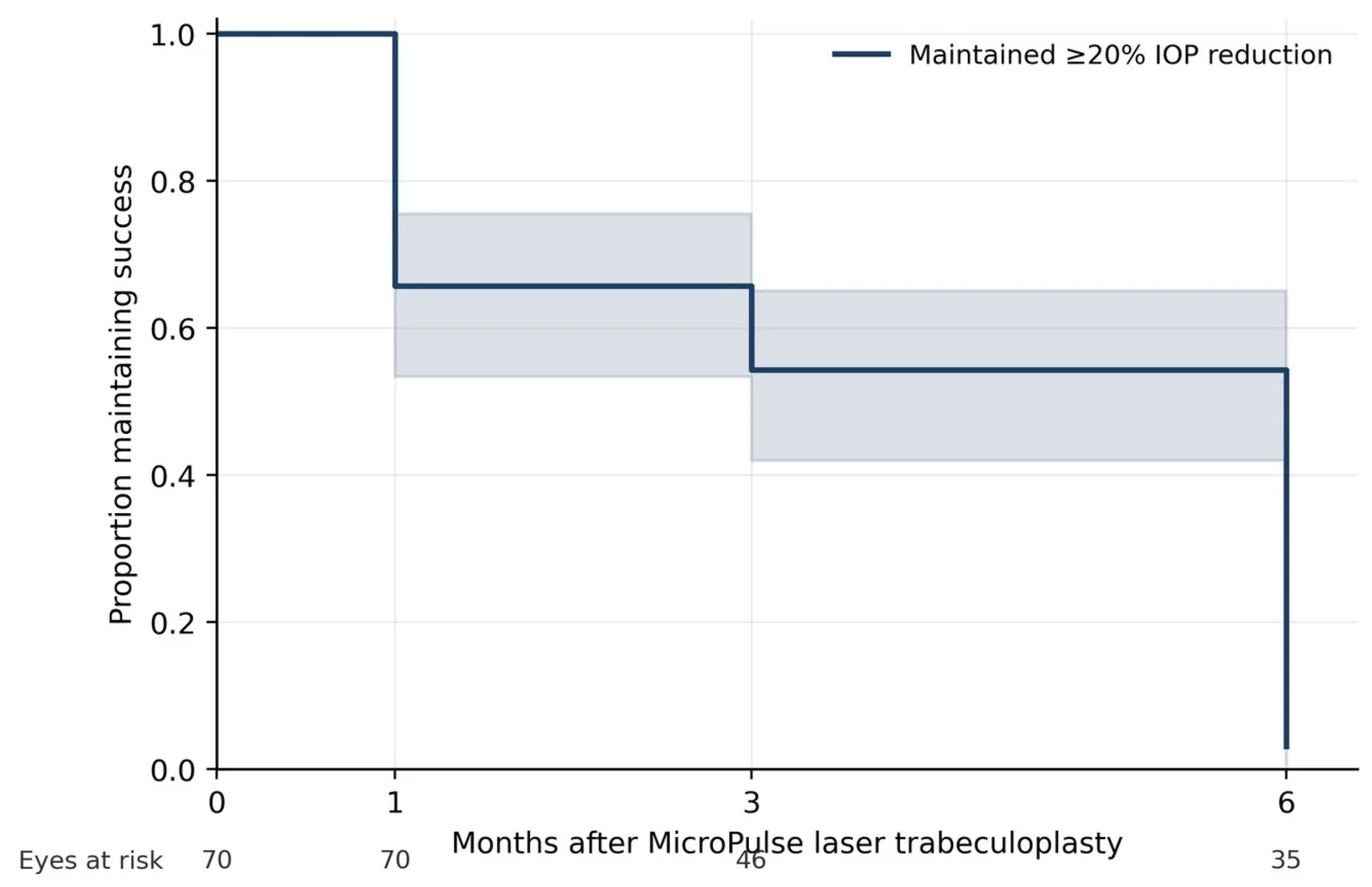
Kaplan–Meier curve for maintenance of treatment success (≥20% IOP reduction from baseline) after MicroPulse laser trabeculoplasty, using all 70 eyes with censoring at the last visit with known status. Shaded band, 95% confidence interval; numbers along the x-axis are eyes at risk entering each interval.

Treatment success at the 20% threshold traced the same arc. Roughly two-thirds of eyes responded early: 62.5% (40/64; 95% CI 50.3–73.3) at one month and 63.0% (34/54; 49.6–74.6) at three months. By six months, only 4.6% (3/65; 1.6–12.7) still qualified. Adding an absolute ceiling of 21 mmHg or less to the definition changed nothing at all: every eye meeting the percentage criterion also met the ceiling at each visit. The durability pattern, in other words, does not hinge on how success is defined. A Kaplan–Meier analysis of maintained success captures the trajectory (Figure 3, Table 3). All 70 eyes entered the survival analysis; 65 failed at an observed visit and five were censored while still successful (three at three months and two at six months, the former having missed the following scheduled visit and the latter censored at the end of follow-up). The proportion holding a ≥20% reduction was 65.7% at one month, 54.3% at three, and 3.1% at six, with most eyes losing the response between the third and sixth month.

An exploratory look tied the early pressure response to baseline IOP, though less strongly than the smaller complete-case subset had suggested. Eyes crossing the 20% threshold at one month had begun from lower pressures than those that did not (25.3 versus 27.0 mmHg, *p* = 0.027). A higher baseline came with a numerically smaller proportional reduction, but the correlation did not reach significance in the full cohort (Spearman rho = −0.20, *p* = 0.11), and the absolute drop did not widen with baseline pressure either (rho = −0.09, *p* = 0.47). The pattern thus runs against, rather than with, regression toward the mean, but it should be read as a weak signal rather than an established gradient. Age carried no predictive value (rho = −0.16, *p* = 0.20), and neither glaucoma subtype nor duty-cycle setting forecast one-month success (Fisher *p* = 0.79 and 0.80).

The corneal endothelium came through largely undisturbed. Cell density held flat over the four visits (2605, 2593, 2602 and 2619 cells/mm^2^ at baseline and 1, 3 and 6 months; every mixed-model change within 10 cells/mm^2^ of baseline, omnibus *p* = 0.170), and neither mean cell area nor central corneal thickness shifted (*p* = 0.237 and 0.910). Two morphological signals did surface. Hexagonality dipped a little at three months (−0.51 percentage points, unadjusted Wilcoxon *p* = 0.021), then came back to baseline. The coefficient of variation in cell size rose slowly and was significantly higher at six months (+0.57, Holm-corrected *p* < 0.001, Cohen’s dz = 0.50).

In absolute terms, these shifts were tiny. The six-month rise in the coefficient of variation ran about 0.6 units and the three-month fall in hexagonality about 0.5 percentage points, both under the device’s repeatability standard deviations (1.6 and 4.9) and comfortably inside its repeatability limits (4.4 and 13.7). Cell density never moved more than 10 cells/mm^2^ at any visit, against a limit near 198, and central corneal thickness changed by less than 1 µm. Once the false discovery rate was controlled across all fifteen corneal comparisons, only the six-month coefficient-of-variation rise held significance (q = 0.004); the three-month hexagonality dip did not (q = 0.16). Equivalence testing settled the matter: at each visit every corneal parameter’s change lay within the device’s repeatability margin (TOST *p* < 0.001 across all twelve comparisons; Table 4). There was margin to spare, as well. The 90% confidence intervals were tight enough that the smallest margin the data would still support ran roughly six to thirty-five times below the device’s repeatability limit. The stricter margin told the same story: swapping each parameter’s repeatability standard deviation (±70.7 cells/mm^2^ for cell density, ±1.6 for the coefficient of variation, ±4.9 percentage points for hexagonality, ±3.4 µm for central corneal thickness) for the wider limit left every parameter equivalent at every visit (TOST *p* < 0.001 throughout). The one morphological signal to survive correction, the six-month coefficient-of-variation rise, showed no sign of being treatment-related: it tracked neither the size of the pressure reduction (Spearman rho = −0.03, *p* = 0.84) nor baseline IOP (rho = 0.13, *p* = 0.30), and did not separate the duty-cycle settings (+0.44 versus +0.64, *p* = 0.59). A laser-driven change should scale with the delivered effect; this one did not. No treatment-related adverse events were recorded over the follow-up.

A complete-case sensitivity analysis restricted to the 47 eyes with all four visits reproduced every one of these findings. IOP changes were −5.30, −5.06 and −1.00 mmHg at one, three and six months against −5.32, −5.00 and −1.02 mmHg in the full cohort; the Friedman test was significant for IOP and the coefficient of variation (both *p* < 0.001) and for hexagonality (*p* = 0.047) but not for cell density, mean cell area or central corneal thickness (*p* = 0.74, 0.90 and 0.88), matching the mixed-model omnibus tests. The direction, magnitude and significance of every conclusion were unchanged, so the added 23 eyes bought precision rather than a different answer.

Since each duty-cycle setting (10% and 15%) belonged to a single surgeon, setting and operator cannot be teased apart. With that caveat, the IOP response held steady across settings at every visit (time-by-duty interaction χ^2^ = 0.85, df = 3, *p* = 0.84); we would not call this a genuine equivalence of the two protocols.

Glaucoma subtype changed little, though these comparisons are exploratory throughout. Primary open-angle and pseudoexfoliation eyes set out from comparable pressures, and the baseline gap in endothelial cell density that a smaller sample had shown was no longer significant once all 70 eyes were included (2570 ± 222 versus 2682 ± 213 cells/mm^2^, *p* = 0.052; Table 1). At every visit mean IOP ran almost identical between them, with mixed-model reductions of −5.31 versus −5.36 mmHg at one month, −5.11 versus −5.22 at three months and −1.04 versus −0.63 at six months for POAG and PEX respectively. No parameter produced a significant time-by-subtype interaction (all unadjusted *p* ≥ 0.09, all Holm-corrected *p* ≥ 0.54). The one interaction that came closest was the coefficient of variation (unadjusted *p* = 0.090; Holm-corrected *p* = 0.54), and its subgroup trajectories differed mainly in shape rather than endpoint: PEX eyes drifted upward earlier (+0.32 at one month and +0.42 at three months, versus +0.12 and +0.11 in POAG eyes) while both groups converged by six months (+0.50 in PEX versus +0.58 in POAG). None of the eighteen visit-wise between-subtype comparisons of change from baseline survived false-discovery-rate correction (all q = 0.95), and all are reported with both unadjusted and adjusted *p* values in Supplementary Table S1. With only 22 pseudoexfoliation eyes these analyses are underpowered, and the absence of a significant interaction should not be read as evidence that the two subtypes behave identically (see Discussion).

## 4. Discussion

Two findings stand out: MLT lowered pressure early but not for long, and no clinically meaningful change in corneal endothelial morphology was detected over six months.

Our IOP curve echoes what Verdina and colleagues reported with the same 577 nm laser: a clear early drop, then a drift back toward baseline by three to six months [9]. Ours followed that shape, with the regression arriving a little later. The figures make the fade tangible; eyes holding a 20% reduction fell from about two-thirds at one month to under 5% at six. None of this is peculiar to MLT. SLT also loses ground, which is why it gets repeated so often and why durability, not the first response, is the clinically important question [3,10,19,20]. In an eye that has never seen a drop, the early result can look impressive, but the realistic expectation is that it will fade and that a second session or a topical agent will be needed before long. One detail sharpens the picture: because no medication was started during follow-up, the six-month pressures reflect the laser alone, not a number propped up by drops. Left to itself, a single MLT session seldom keeps pressure a fifth below baseline past the first quarter.

Whether this steep regression generalizes is unclear, and the missing severity data bear on the question. Because severity was assessed inconsistently across the cohort—visual fields in some patients, retinal nerve fiber layer imaging in others, and neither uniformly across visits—we could not derive a consistent staging measure, so we cannot tell whether the quick loss of effect reflects more advanced disease, heavier baseline trabecular dysfunction, or just the natural course of one low-energy laser session in untreated eyes. The honest reading is that these six-month numbers describe this cohort rather than fix a value for MLT at large; teasing the explanations apart would need a staged, prospective comparison.

The endothelium held up. Cell density, mean cell area, and corneal thickness stayed flat across all four visits, in line with MLT and SLT series that reported no cell loss [9,14]. Two measures, the coefficient of variation and hexagonality, did cross the significance line, which is easy enough to do with paired data collected at four visits in seventy eyes. Their magnitudes, though, were trivial. Family-wide correction left only the six-month coefficient-of-variation change standing, and formal equivalence testing placed every corneal change, at every visit, inside the device’s repeatability margin (Table 4). That six-month shift amounted to roughly 0.6 units, under the instrument’s repeatability standard deviation of 1.6 and nowhere near its 4.4 limit; the hexagonality dip fell well within the same band [15]. Detectable on paper, then, but far too small to mark structural injury at this point. We do not treat these as established remodeling. Even so, the six-month coefficient-of-variation change carried a moderate effect size (Cohen’s dz = 0.50) and was the single signal to outlast family-wide correction, so a slow, low-grade rise in polymegethism cannot be ruled out and deserves watching past six months. It also helps that the two parameters that moved at all are the least reproducible specular measurements on Topcon systems [17,18], which makes a small apparent shift in either easier to pin on measurement variability than on biology. The same brief wobble in polymegethism and pleomorphism appears after SLT and settles within weeks [12,13].

The subtype comparison deserves more caution than a bare interaction *p* value conveys. Pseudoexfoliation is not a variant of primary open-angle glaucoma but a distinct systemic fibrillopathy, in which fibrillar material accumulates in the juxtacanalicular meshwork alongside heavy, blotchy trabecular pigmentation. That combination is usually said to produce a more volatile clinical course: higher and more fluctuating untreated pressures, a greater propensity to post-laser pressure spikes as pigment and debris are liberated into an already loaded outflow pathway, faster structural progression, and a shorter-lived response to trabeculoplasty. On the endothelial side, exfoliation material and the associated pigment dispersion are toxic to the corneal endothelium, and pseudoexfoliation corneas show lower cell counts, greater polymegethism, and reduced functional reserve even before any intervention. On prior grounds, then, one would expect pseudoexfoliation eyes to respond differently from primary open-angle eyes, not identically.

We found no such difference, but the study was not built to detect one. With 22 pseudoexfoliation eyes against 48 primary open-angle eyes, and with attendance thinning the interim visits further, the interaction tests carried little power: a difference of even moderate size between the two subtype trajectories would have been missed more often than not, and every interaction was estimated with a confidence interval wide enough to accommodate clinically relevant divergence in either direction. The non-significant interaction terms are therefore a Type II error waiting to happen rather than evidence of equivalence, and we do not present them as showing that the subtypes behave alike. Two observations reinforce that reading. First, the coefficient of variation, the one parameter with any hint of subtype dependence, produced the smallest interaction *p* value of the six (unadjusted *p* = 0.090) and a subgroup pattern in which pseudoexfoliation eyes drifted upward earlier than primary open-angle eyes before the two converged at six months. That is exactly the shape one would predict if pigment-laden, exfoliation-affected corneas were marginally more perturbed by the laser, and it is precisely the sort of small early signal that an underpowered interaction test cannot resolve. It is also, on the same evidence, entirely consistent with noise, and it did not survive correction across the six interactions or across the eighteen visit-wise comparisons (all q = 0.95). We flag it as a hypothesis for a properly powered study, not as a finding. Second, our pseudoexfoliation eyes started with a numerically higher endothelial cell density than the primary open-angle group (2682 versus 2570 cells/mm^2^), the reverse of what the toxicity of exfoliation material would predict. In the smaller complete-case subset this gap reached significance; across the full cohort it did not (*p* = 0.052), which is itself instructive about how readily a modest subgroup imbalance can look like a finding. Sampling variation in a modest, single-center series is the likelier explanation than a real biological reversal, and settling the question would take a larger, prospectively stratified sample with severity staging.

The mechanism is consistent with the overall picture. Micropulse mode splits the energy into short bursts with cooling gaps, so the meshwork takes on little heat, and little energy scatters forward to the cornea [5,7]. At 1000 mW through a 300 µm spot over 360°, the dose reaching the endothelium is modest, and the flat density and thickness curves fit that. Still, the endothelium cannot replace lost cells [11], so even a slow, low-grade insult would count over time; following these eyes past six months would be worthwhile, both to confirm the cornea stays stable and to chart how quickly the pressure effect decays.

The two duty-cycle settings produced similar pressure curves, but we treat that comparison with caution: a different surgeon used each setting, fully confounding setting with operator, and the analysis was underpowered. At best it hints that the lower-energy setting was not obviously worse; it is not evidence that the protocols are equivalent.

Several limitations temper these conclusions. The design was retrospective and single-arm, with no control group, so the natural drift of endothelial measurements over time cannot be separated from a treatment effect, even though the parameters that mattered most stayed flat. This is the reason we frame the corneal result as the absence of a detectable, clinically meaningful change over six months rather than as a demonstration of endothelial safety; the latter would require a control arm and longer follow-up. Attendance at the interim visits was incomplete. We addressed this by analyzing all 70 eyes with mixed models that use every recorded observation rather than by deleting patients, and by showing that those who attended all four visits did not differ at baseline from those who missed one (Supplementary Table S2); the complete-case analysis reproduced every finding. Missingness that depends on unmeasured factors cannot be excluded, but the intermittent pattern of the missed visits, and the fact that 93% of eyes returned at six months, make a strongly informative mechanism unlikely. With the first scheduled visit at one month, immediate post-laser events such as transient IOP spikes went uncaptured, which matters particularly for the pseudoexfoliation eyes in whom such spikes are most expected. The cohort was modest and single-center, follow-up ended at six months, and the only laser parameter that varied between patients was the duty cycle (10% versus 15%), set by surgeon preference and without measurable effect on the IOP response; since each surgeon kept to one setting, duty cycle and operator are confounded. Our equivalence margins came from the device’s repeatability in a separate, non-glaucomatous cohort and were not re-derived here; because repeatability may differ in glaucomatous and pseudoexfoliation corneas, those margins are best taken as external reference bounds rather than population-specific thresholds. Optical coherence tomography of the optic nerve head supported the baseline diagnosis, but severity assessment was heterogeneous—some patients were evaluated functionally with visual fields and others structurally with retinal nerve fiber layer imaging—and was not obtained uniformly for every patient or repeated at each visit, so a consistent baseline severity measure could not be derived or linked to the pressure response. All subtype comparisons were exploratory and underpowered, and are reported as such. Longer comparative studies, ideally against SLT, prospectively stratified by subtype, and with endothelial imaging carried past a year, would clarify the durability of the pressure effect, the meaning of the polymegethism signal, and whether pseudoexfoliation eyes really do behave like primary open-angle eyes after MLT.

## 5. Conclusions

In treatment-naïve glaucoma, 577 nm MLT trimmed IOP by about a fifth through the first three months, then largely surrendered it. Across the same span no endothelial parameter crossed the device’s measurement error, and equivalence testing agreed that no clinically meaningful change had occurred. Allowing for a retrospective, single-arm design and six months of follow-up, we detected no meaningful endothelial change over the study period, while the pressure benefit is a starting point rather than a destination and will often need reinforcing.

## Figures and Tables

**Table 1:** Baseline characteristics of the study cohort, overall and stratified by glaucoma subtype.

Characteristic	All (n = 70)	POAG (n = 48)	PEX (n = 22)	*p*
Age, years (mean ± SD)	59.1 ± 5.3	59.0 ± 5.6	59.4 ± 4.4	0.780
Age range, years	47–72	47–72	49–69	—
Sex, women/men	35/35	26/22	9/13	0.440
Eye, right/left	36/34	27/21	9/13	0.310
Duty cycle, 15%/10%	39/31	27/21	12/10	1.000
Systemic hypertension/ goitre/none	35/4/31	23/3/22	12/1/9	0.860
Baseline IOP, mmHg	25.9 ± 2.5	25.9 ± 2.6	25.8 ± 2.3	0.924
Baseline endothelial cell density, cells/mm^2^	2605 ± 224	2570 ± 222	2682 ± 213	0.052
Baseline mean cell area, µm^2^	386.3 ± 33.2	391.6 ± 33.7	374.7 ± 29.5	0.051
Baseline hexagonality, %	52.4 ± 4.5	52.2 ± 4.3	52.6 ± 5.1	0.517
Baseline coefficient of variation, %	33.3 ± 1.7	33.5 ± 1.7	32.9 ± 1.7	0.127
Baseline central corneal thickness, µm	539.8 ± 18.2	540.5 ± 18.8	538.3 ± 17.4	0.653

Values are mean ± SD or n. IOP, intraocular pressure; POAG, primary open-angle glaucoma; PEX, pseudoexfoliation glaucoma; SD, standard deviation. Continuous variables compared by Mann–Whitney U test, categorical variables by Fisher’s exact or chi-square test. All eyes were treatment-naïve with open angles (Shaffer grade 3–4). Subtype comparisons are exploratory and unadjusted for multiplicity.

**Table 2:** Intraocular pressure and corneal endothelial parameters at baseline and at 1, 3, and 6 months after MicroPulse laser trabeculoplasty, with mixed-model changes from baseline.

Parameter (Unit)	Baseline	1 Month	3 Months	6 Months	Mixed-Model Change from Baseline, Mean (95% CI); Omnibus *p*
Intraocular pressure (mmHg)	25.9 ± 2.5 (70)	20.6 ± 3.9 (64)	21.0 ± 4.0 (54)	24.8 ± 3.2 (65)	1 M: −5.32 (−5.97, −4.67); 3 M: −5.00 (−5.69, −4.31); 6 M: −1.02 (−1.67, −0.37); *p* < 0.001
Endothelial cell density (cells/mm^2^)	2605 ± 224 (70)	2593 ± 214 (64)	2602 ± 226 (56)	2619 ± 213 (63)	1 M: +6.31 (−9.96, +22.58); 3 M: −9.41 (−26.45, +7.64); 6 M: +9.12 (−7.21, +25.44); *p* = 0.170
Mean cell area (µm^2^)	386.3 ± 33.2 (70)	387.9 ± 32.1 (64)	386.8 ± 33.5 (56)	384.4 ± 30.8 (63)	1 M: −1.01 (−3.72, +1.70); 3 M: +1.48 (−1.36, +4.31); 6 M: −1.31 (−4.03, +1.41); *p* = 0.237
Hexagonality (%)	52.4 ± 4.5 (70)	52.5 ± 4.9 (64)	52.2 ± 4.6 (56)	52.2 ± 5.3 (63)	1 M: −0.11 (−0.54, +0.31); 3 M: −0.51 (−0.96, −0.06); 6 M: +0.03 (−0.39, +0.46); *p* = 0.083
Coefficient of variation (%)	33.3 ± 1.7 (70)	33.5 ± 1.6 (64)	33.4 ± 1.9 (56)	33.8 ± 2.0 (63)	1 M: +0.17 (−0.08, +0.41); 3 M: +0.17 (−0.09, +0.43); 6 M: +0.57 (+0.32, +0.82); *p* < 0.001
Central corneal thickness (µm)	539.8 ± 18.2 (70)	539.5 ± 17.2 (64)	539.5 ± 19.4 (56)	540.7 ± 19.9 (63)	1 M: −0.33 (−1.71, +1.04); 3 M: −0.17 (−1.61, +1.27); 6 M: +0.18 (−1.20, +1.56); *p* = 0.910

Values are mean ± SD, with the number of eyes examined at that visit in parentheses. Change from baseline was estimated by linear mixed models with a random intercept per patient, fitted to all available observations from all 70 eyes; CI, confidence interval. The omnibus p value is a Wald test of the three visit contrasts jointly.

**Table 3:** Cross-sectional and Kaplan–Meier estimates of treatment success (≥20% reduction in intraocular pressure) after MicroPulse laser trabeculoplasty.

Visit	Eyes Meeting ≥ 20% Reduction, n/N (%; 95% CI)	Kaplan–Meier Survival of Success, % (95% CI)	Eyes at Risk Entering the Interval
1 month	40/64 (62.5; 50.3–73.3)	65.7 (53.4–75.5)	70
3 months	34/54 (63.0; 49.6–74.6)	54.3 (42.0–65.1)	46
6 months	3/65 (4.6; 1.6–12.7)	3.1 (0.6–9.6)	35

Success defined a priori as ≥20% reduction in IOP from baseline. Cross-sectional success counts eyes meeting the threshold among those examined at that visit (percentage confidence intervals are Wilson intervals); the denominator therefore varies with attendance. Kaplan–Meier survival counts eyes that maintained success continuously from treatment (first observed failure), with eyes still successful at their last attended visit censored at that visit. Of the 70 eyes, 65 failed and five were censored. Because status was assessed only at the three scheduled visits, failure times are interval-censored and the estimates track visit times rather than continuous follow-up.

**Table 4:** Equivalence testing of corneal endothelial changes against the device’s repeatability limits.

Parameter	Largest Change (Visit)	90% CI	Equivalence Margin (±)	TOST *p*	Conclusion
Endothelial cell density (cells/mm^2^)	+9.70 (6 M)	−6.29, +25.69	197.9	<0.001	Equivalent
Coefficient of variation	+0.56 (6 M)	+0.32, +0.79	4.4	<0.001	Equivalent
Hexagonality (pp)	−0.54 (3 M)	−0.90, −0.17	13.7	<0.001	Equivalent
Central corneal thickness (µm)	−0.31 (1 M)	−1.18, +0.56	9.6	<0.001	Equivalent

TOST, two one-sided tests, computed on all available baseline-to-visit pairs. The equivalence margin is the device’s repeatability limit for each parameter; equivalence also held when the stricter repeatability standard deviation was used as the margin (TOST *p* < 0.001 for all). Mean cell area, being the reciprocal of endothelial cell density, is not tested separately here but was included in the false-discovery-rate correction across all fifteen corneal comparisons. The largest change across the three visits is shown; equivalence held at every visit for all parameters. pp, percentage points.

## Data Availability

The data supporting the findings are available from the corresponding author on reasonable request.

## Supplementary Materials

The following supporting information can be downloaded at: https://www.mdpi.com/article/10.3390/life16081237/s1, Table S1: Change from baseline in intraocular pressure and corneal endothelial parameters by glaucoma subtype, with time-by-subtype interaction tests and both unadjusted and false-discovery-rate-adjusted *p* values. Table S2: Baseline characteristics of patients with complete four-visit follow-up versus those missing at least one visit. Table S3: Complete-case sensitivity analysis (n = 47) versus the primary all-available-data analysis (n = 70).

## Abbreviations

MLT	MicroPulse laser trabeculoplasty
IOP	intraocular pressure
SLT	selective laser trabeculoplasty
POAG	primary open-angle glaucoma
PEX	pseudoexfoliation glaucoma
MAR	missing at random
SD	standard deviation
CI	confidence interval
TOST	two one-sided tests
FDA	U.S. Food and Drug Administration
