# Supplementary material for: Corneal Endothelial Safety and Intraocular Pressure Outcomes of MicroPulse Laser Trabeculoplasty in Treatment-Naïve Glaucoma: A Six-Month Retrospective Study
Source: Life (Basel). 2026 Jul 27;16(8):1237. doi: 10.3390/life16081237 (PMC13514978; doi:10.3390/life16081237)
Supplement: Supplementary file 1 [file life-16-01237-s001.zip › life-4442973-supplementary.pdf]

Supplementary Materials

Corneal Endothelial Safety and Intraocular Pressure Outcomes of MicroPulse Laser Trabeculoplasty in Treatment-Naïve Glaucoma: A Six-Month Retrospective Study

Table S1. Change from baseline by glaucoma subtype, with interaction tests and multiplicity adjustment

Parameter / visit	n POAG / PEX	POAG change, mean (95% CI)	PEX change, mean (95% CI)	p (unadj.)	q (BH)	Interaction p
**IOP (mmHg)**						
1 month	42 / 22	−5.31 (−6.08, −4.54)	−5.36 (−6.51, −4.21)	0.954	0.954	0.988 (Holm 1.00)
3 months	36 / 18	−5.11 (−5.83, −4.40)	−5.22 (−6.38, −4.06)	0.435	0.950	
6 months	46 / 19	−1.04 (−1.80, −0.29)	−0.63 (−1.66, +0.40)	0.576	0.950	
**Endothelial cell density (cells/mm²)**						
1 month	42 / 22	+5.48 (−6.63, +17.58)	+12.23 (−9.40, +33.86)	0.543	0.950	0.667 (Holm 1.00)
3 months	37 / 19	−14.35 (−33.77, +5.06)	+1.47 (−20.74, +23.69)	0.377	0.950	
6 months	45 / 18	+11.87 (−13.21, +36.94)	+4.28 (−16.17, +24.73)	0.873	0.950	
**Mean cell area (μm²)**						
1 month	42 / 22	−0.95 (−3.01, +1.10)	−1.91 (−4.79, +0.97)	0.557	0.950	0.627 (Holm 1.00)
3 months	37 / 19	+2.35 (−0.83, +5.53)	−0.26 (−3.44, +2.91)	0.363	0.950	
6 months	45 / 18	−1.89 (−6.29, +2.51)	−0.28 (−3.07, +2.51)	0.897	0.950	
**Hexagonality (%)**						
1 month	42 / 22	−0.19 (−0.66, +0.28)	+0.23 (−0.55, +1.00)	0.427	0.950	0.697 (Holm 1.00)

Parameter / visit	n POAG / PEX	POAG change, mean (95% CI)	PEX change, mean (95% CI)	p (unadj.)	q (BH)	Interaction p
3 months	37 / 19	−0.57 (−1.13, −0.00)	−0.47 (−1.12, +0.17)	0.777	0.950	
6 months	45 / 18	−0.11 (−0.61, +0.38)	+0.11 (−0.61, +0.84)	0.510	0.950	
**Coefficient of variation (%)**						
1 month	42 / 22	+0.12 (−0.17, +0.40)	+0.32 (−0.10, +0.73)	0.557	0.950	0.090 (Holm 0.54)
3 months	37 / 19	+0.11 (−0.18, +0.40)	+0.42 (+0.04, +0.80)	0.195	0.950	
6 months	45 / 18	+0.58 (+0.22, +0.93)	+0.50 (+0.10, +0.90)	0.780	0.950	
**Central corneal thickness (μm)**						
1 month	42 / 22	−0.36 (−1.66, +0.95)	−0.23 (−1.89, +1.43)	0.831	0.950	0.755 (Holm 1.00)
3 months	37 / 19	−0.43 (−2.23, +1.37)	+0.16 (−2.95, +3.27)	0.709	0.950	
6 months	45 / 18	+0.38 (−1.10, +1.85)	−0.67 (−3.29, +1.96)	0.664	0.950	

Change from baseline computed on all available baseline-to-visit pairs within each subtype; n gives the number of contributing pairs. The unadjusted p value compares the change between subtypes at that visit (Mann–Whitney U); q is the Benjamini–Hochberg false-discovery-rate-adjusted value across all eighteen visit-wise comparisons. The interaction p value is a Wald test of the time-by-subtype interaction terms within the linear mixed model for that parameter, shown once per parameter, with the Holm-adjusted value across the six parameters in parentheses. All analyses in this table are exploratory. POAG, primary open-angle glaucoma; PEX, pseudoexfoliation glaucoma; IOP, intraocular pressure; CI, confidence interval.

Table S2. Baseline characteristics of patients with complete four-visit follow-up versus those missing at least one visit

Characteristic	All four visits (n = 47)	≥1 visit missed (n = 23)	p
Age, years	59.3 ± 5.1	58.7 ± 5.6	0.594
Sex, women / men	24 / 23	11 / 12	1.000
Eye, right / left	24 / 23	12 / 11	1.000
Glaucoma subtype, POAG / PEX	32 / 15	16 / 7	1.000
Duty cycle, 15% / 10%	26 / 21	13 / 10	1.000
Baseline IOP, mmHg	26.0 ± 2.5	25.5 ± 2.5	0.565

Characteristic	All four visits (n = 47)	≥1 visit missed (n = 23)	p
Baseline endothelial cell density, cells/mm²	2605 ± 223	2606 ± 231	0.950
Baseline mean cell area, μm²	386.2 ± 33.1	386.5 ± 34.0	0.925
Baseline hexagonality, %	52.6 ± 4.3	51.8 ± 5.0	0.440
Baseline coefficient of variation, %	33.3 ± 1.7	33.3 ± 1.8	0.974
Baseline central corneal thickness, μm	540.0 ± 17.9	539.4 ± 19.3	0.881

Values are mean ± SD or n. Continuous variables compared by Mann–Whitney U test, categorical variables by Fisher's exact test. No baseline variable differed between the two groups, supporting the missing-at-random assumption used in the primary mixed-model analysis. IOP, intraocular pressure; POAG, primary open-angle glaucoma; PEX, pseudoexfoliation glaucoma; SD, standard deviation.

Table S3. Complete-case sensitivity analysis (n = 47) versus the primary all-available-data analysis (n = 70)

Parameter	Change at 6 months, n = 70 (primary)	Change at 6 months, n = 47 (complete case)	Friedman p (n = 47)
Intraocular pressure (mmHg)	−1.02 (−1.67, −0.37)	−1.00 (−1.75, −0.25)	<0.001
Endothelial cell density (cells/mm²)	+9.12 (−7.21, +25.44)	+9.40 (−10.24, +29.04)	0.736
Mean cell area (μm²)	−1.31 (−4.03, +1.41)	−1.21 (−4.44, +2.02)	0.896
Hexagonality (%)	+0.03 (−0.39, +0.46)	−0.09 (−0.54, +0.37)	0.047
Coefficient of variation (%)	+0.57 (+0.32, +0.82)	+0.66 (+0.41, +0.91)	<0.001
Central corneal thickness (μm)	+0.18 (−1.20, +1.56)	−0.32 (−1.85, +1.21)	0.884

Mixed-model mean change from baseline at six months with 95% confidence intervals. The Friedman test requires complete data and is therefore reported only for the complete-case subset, as a distribution-free check on the omnibus time effect. Every conclusion is identical between the two analyses.
